# Experimental Investigation of the Rheological Behavior of an Oil-Based Drilling Fluid with Rheology Modifier and Oil Wetter Additives

**DOI:** 10.3390/molecules26164877

**Published:** 2021-08-12

**Authors:** Mobeen Murtaza, Sulaiman A. Alarifi, Muhammad Shahzad Kamal, Sagheer A. Onaizi, Mohammed Al-Ajmi, Mohamed Mahmoud

**Affiliations:** 1 Department of Petroleum Engineering, King Fahd University of Petroleum & Minerals (KFUPM), Dhahran 31261, Saudi Arabia; mobeen@kfupm.edu.sa (M.M.); mmahmoud@kfupm.edu.sa (M.M.); 2 Center for Integrative Petroleum Research (CIPR), King Fahd University of Petroleum & Minerals, Dhahran 31261, Saudi Arabia; shahzadmalik@kfupm.edu.sa; 3 Department of Chemical Engineering, King Fahd University of Petroleum & Minerals, Dhahran 31216, Saudi Arabia; onaizi@kfupm.edu.sa; 4 Petroleum & Energy Logistics and Services Co., Al-Khobar 34227, Saudi Arabia; mohammed.ajmi@petrogistix.com

**Keywords:** drilling fluids, oil-based drilling fluid, flat rheology, rheology modifier, oil wetter

## Abstract

Drilling issues such as shale hydration, high-temperature tolerance, torque and drag are often resolved by applying an appropriate drilling fluid formulation. Oil-based drilling fluid (OBDF) formulations are usually composed of emulsifiers, lime, brine, viscosifier, fluid loss controller and weighting agent. These additives sometimes outperform in extended exposure to high pressure high temperature (HPHT) conditions encountered in deep wells, resulting in weighting material segregation, high fluid loss, poor rheology and poor emulsion stability. In this study, two additives, oil wetter and rheology modifier were incorporated into the OBDF and their performance was investigated by conducting rheology, fluid loss, zeta potential and emulsion stability tests before and after hot rolling at 16 h and 32 h. Extending the hot rolling period beyond what is commonly used in this type of experiment is necessary to ensure the fluid’s stability. It was found that HPHT hot rolling affected the properties of drilling fluids by decreasing the rheology parameters and emulsion stability with the increase in the hot rolling time to 32 h. Also, the fluid loss additive’s performance degraded as rolling temperature and time increased. Adding oil wetter and rheology modifier additives resulted in a slight loss of rheological profile after 32 h and maintained flat rheology profile. The emulsion stability was slightly decreased and stayed close to the recommended value (400 V). The fluid loss was controlled by optimizing the concentration of fluid loss additive and oil wetter. The presence of oil wetter improved the carrying capacity of drilling fluids and prevented the barite sag problem. The zeta potential test confirmed that the oil wetter converted the surface of barite from water to oil and improved its dispersion in the oil.

## 1. Introduction

Drilling fluids are essential for a variety of reasons, including carrying drilled cuttings to the surface, cleaning, cooling and lubricating the drilling bit, reducing friction and maintaining borehole integrity [1,2,3,4,5]. Using the appropriate drilling fluid is a crucial part of any successful drilling operation. The drilling fluid’s cost, environmental impact and shale inhibition characteristics are all taken into account in selecting the optimum drilling fluid [6,7,8,9]. Oil-based and water-based drilling fluids are two main types of drilling fluids used in oil and gas drilling operations that differ in use and composition. Water-based drilling fluids (WBDFs) are less expensive and better for the environment; however, they have limits in high pressure high temperature (HPHT) wells and shale bearing formations. Although OBDFs are not frequently used for drilling operations mainly due to environmental challenges [10,11,12], they are recommended for wellbore stability in HPHT wells and shale-bearing formations. The use of OBDFs is becoming more widespread in shale gas exploration and high temperature deep wells due to their superior shale inhibition characteristics, lubricity and stability at high temperatures when compared with WBDF [13,14]. When it comes to shale formations, OBDF is the optimal choice as hydration and swelling of the water-sensitive shale would be caused by the water contents in the WBDF [15,16].

The required properties for drilling fluids are described in the API I3B-2 procedures of standard practice for testing oil-based drilling fluids [17]. In OBDFs, high-density weighting elements such as barium sulfate, hematite, manganese tetroxide and ilmenite are added to increase the weight of the drilling fluids. API-grade barite has remained the standard weighting material in the drilling fluid among these weight materials. It can be easily processed to a particle size that lowers settling and reduces shaker screen losses. Its chemical inertness permits it to be utilized in a variety of drilling fluids with various chemical components. The hydrophilic solid particles of barite and solid cuttings tend to combine with water and coalesce, causing high viscosity and segregation. Also, destroying the stability of drilling fluid, making the system difficult to maintain. Therefore, posing a significant well control risk [18]. Particles that have settled obstruct drilling and production operations [19].

Additionally, accumulated solid particles and drilled cuttings increase the likelihood of pipe sticking and reduce penetration rate [20]. Barite sag occurs in both vertical and directional wells under static and dynamic conditions but is more severe under dynamic conditions with a low shear rate. Barite sag in OBDF is influenced by the properties of the drilling fluid and drilling parameters such as flow rate, hole geometry, and pipe trips. Among these, drilling fluid rheology has garnered the most attention and is widely regarded as a significant and complex factor affecting barite sag [21]. Several researchers prevented the barite sag by modifying the rheology of drilling fluids [22,23,24,25]. Another strategy for minimizing weight-material sag in drilling fluids is to use finer-particle-size weight materials. In some studies, barite and other heavy-weight materials were mixed to reduce the barite sag. All mentioned solutions result in an undesirable increase in rheological properties.

Drilling fluids generally behave as non-Newtonian fluids and their behavior varies depending on the temperature and pressure. The temperature shows a prominent effect on the rheology of the drilling fluids. Additionally, the rheology of drilling fluid varies significantly with pressure and temperature, which creates complications when drilling deep wells. The viscosity of the drilling fluids can increase severalfold when the temperature of the fluid is reduced from ambient to seabed conditions (4 °C in the Gulf of Mexico). Similarly, it can plummet severalfold because of increasing the temperature of the bottomhole from ambient conditions. Such a change in viscosity can have significant consequences on the equivalent circulating density (ECD), the stability of the wellbore, and the suspension properties [26]. The effect of temperature reduction on the rheological properties of Jatropha curcas oil, groundnut oil and diesel oil was studied by Anawe et al. [27]. When the temperature was increased, the viscosities of all the drilling fluids steadily decreased. At temperatures ranging from 38 °C (100 °F) to 316 °C (600 °F) and pressures ranging from 34.5 MPa (5000 psi) to 172.4 MPa (25000 psi), Amani investigated the impact of high-temperature high-pressure on the rheological properties of oil and water-based drilling fluids. When the temperature was raised from 38 °C (100 °F) to 204 °C (400 °F), the rheological characteristics of the oil-based drilling fluid decreased, then increased when the temperature was raised from 204 °C (400 °F) to 316 °C (600 °F) [28].

Dardir et al. evaluated the rheological characteristics of a formulated ester-based drilling fluid and original field ester-based drilling fluid at temperatures ranging from 25 °C (77 °F) to 100 °C (212 °F), as well as under aging conditions of 16 h at 177 °C (350 °F) temperature. The rheological characteristics of the formulated ester and field ester-based drilling fluids decreased as the temperature increased from 25 °C (77 °F) to 100 °C (212 °F). Further, the rheological properties of the drilling fluids degraded as the aging time increased from 0 to 16 h at high temperature of 177 °C (350 °F) [29].

Drilling complications are expected to occur during difficult drilling conditions, such as drilling through shale layers or rock layers with different hardnesses. These drilling complications could result in a stuck pipe, shale swelling or wellbore instability [30,31,32,33,34,35,36]. Although OBDF is often preferred for its superior performance in drilling a difficult well because it can easily combat these drilling complications, there is still a need for additives that are more stable physically or chemically for a longer period under extreme temperature or pressure conditions [37]. The additives play a major role in ensuring the OBDF is maintaining certain rheological and fluid loss properties to ensure its effectiveness. Table 1 summaries the main recommended rheological and fluids loss properties of OBDF.

Over the years, surfactants are being used for several applications in drilling fluids. In OBDF, the most common applications of surfactants are as emulsifiers and wetting agents [41]. As barite is difficult to disperse in oil, it must be converted from a water-wetted to an oil-wetted surface. The use of an external wetting agent (oil wetter) causes the barite and drilling cuttings particle surfaces that have just entered an oil-based drilling fluid to swiftly transform to a lipophilic, ensuring that they can be suspended in the oil phase and make stable emulsion. Developing drilling fluids that contain polymer has been a known practice since the 1950s as polymer mud was initially developed to improve the drilling efficiency [42]. Polymer-based drilling fluids can increase penetration rates, improve core recovery, minimize formation damage and retain near-gauge hole [43]. Using a polymer as a rheology modifier in OBDF showed improvement in its shearing force under high temperature and high-pressure conditions and improved stability [44].

This study’s objective is to test the performance of different oil-based drilling fluids (OBDFs) under high pressure high temperatures. Four different formulations of OBDFs (DF-1, DF-2, DF-3 and DF-4) were tested in this study. The objective is to investigate how the different formulations of the OBDF will behave in terms of their density, rheology and stability. Ultimately, this work investigated the drilling fluid’s capability to provide flat rheology properties under high temperature and pressure conditions. Furthermore, the effects of hot rolling the drilling fluids for different extended periods (16 h and 32 h) were investigated too. The original formulation of the drilling fluid (DF-1) is a barite-free OBDF. Barite was used as a weighting material in the other two formulations (DF-2 and DF-3). Rheology modifier and oil wetter are both included in DF-3 and DF-4. DF-4 formulation was slightly changed from DF-3. To test the stability of the DF-2, DF-3 and DF-4 formulations, they were hot-rolled for 16 to 32 h. Several experiments were conducted to assess the performance of drilling fluids, including density, rheology, fluid loss and electrical stability.

## 2. Materials and Method

### 2.1. Materials

#### 2.1.1. Oil-Based Drilling Fluid Preparation

The OBDF was prepared as per API standard procedure (API-RP-13I) [45]. A Hamilton beach mixer was used to mix different components of the drilling fluid formulation. First, primary and secondary emulsifiers were mixed in a Diesel oil. Subsequently, lime and calcium chloride brine solutions were mixed for different mixing times. Then organophilic clay and organophilic lignite were added as viscosifier and as fluid loss control additives, respectively. Finally, calcium carbonate was added, acting as a bridging agent. Two alternative drilling fluids formulations (DF-2 and DF-3) and the original barite-free drilling fluid formulation (DF-1) were prepared. For DF-2, barite has been added as a weighting material. For DF-3, along with the addition of barite, rheology modifier and oil wetter were mixed too. DF-4 was mixed based on the performance of the DF-3 and was slightly modified from DF-3. DF-4 contained high concentrations of fluid loss controller and oil wetter. The water content was also reduced in DF-4. The formulation and the additives used with their mixing time for the four OBDFs formulations (DF-1, DF-2, DF-3 and DF-4) are summarized in Table 2. The three alternative formulations of OBDFs were tested and compared in this paper.

To test the stability of the DF-2 and DF-3 formulations, they were both hot-rolled (aging) at 177 °C (350 °F) temperature using a roller oven for 16 h (DF-2-16h and DF-3-16h) and for 32 h along with DF-4 (DF-2-32h, DF-3-32h and DF-4-32h). All the tests in this work were conducted on the three different formulations of OBDF drilling fluids (DF-1, DF-2 and DF-3) and conducted on the hot-rolled DF-2-16h, DF-3-16h, DF-2-32h and DF-3-32h drilling fluids as they are compared with the OBDF formulations before hot rolling. Furthermore, DF-4 formulation was tested after hot rolling for 32 h at high temperature conditions.

#### 2.1.2. Oil-Based Drilling Fluid Additives

The term “flat rheology” refers to drilling fluids, a concept born out of the desire to eliminate drilling mud loss during casing and liner operations in deepwater wells. It was believed that if a drilling fluid had a “near” constant yield point at low temperature and high pressure and increased temperature and high pressure, accuracy in predicting pressure response behavior during pipe tripping would improve, thereby eliminating or minimizing downhole losses. This is a concept worth developing further. As a result, drilling fluid manufacturers focused their efforts on the flat system. However, the ability to control a flat rheology profile is influenced by several non-controllable mechanisms, including temperature and pressure variations, rheological modifiers and drill solids, changing shear rates in the annulus and variations in the annulus alkalinity and changes in rheological modifier concentration. The superior performance is achieved by combining the appropriate emulsifiers, wetting agents, rheology modifiers and supplementary viscosifiers. A simplified and improved flat rheology system enhances emulsion stability as well as thermal and fluid lubricity.

In this study, OBDFs were prepared by combining various emulsifiers, viscosifiers, wetting agents, rheology modifiers, fluid loss controllers and weighting agents to achieve the desired properties such as flat rheology and high temperature tolerance at extended hot rolling time. The primary emulsifier used is a fatty acid amide and the secondary emulsifier used is a fatty acid polyamide. These emulsifiers are a hydrocarbon-based, non-ionic wetting, demulsified surfactant, which acts on oil-water interface to prevent emulsions and generates a stable micro emulsion in water. They are designed for general use in the removal of hydrocarbons and oil-based products. Besides, they are used to clean metal surfaces. They are compatible with all cementation and stimulation additives. The viscosifier is a treated organoclay which is a gelling agent used to obtain high viscosity values. The effect of these additives in the rheology profile is tested after hot rolling for at least 16 h to observe its effect in obtaining a flat rheology profile. The fluid loss controller used is a quaternary Amine treated organophilic lignite. The rheology modifier used is a polymerized urea- formaldehyde compound with α-cellulose filler. The oil wetter additive used is an alcohol alkoxylate high-performance polymeric additive, specifically developed to control fluid loss and as a result it outperforms traditional additives. The product works well at temperatures up to 150 °C.

### 2.2. Methodology

#### 2.2.1. Density and Oil/Water Contents Measurement

Drilling fluid density affects the application of hydrostatic pressure and the prevention of formation’s fluid incursion into the wellbore. In this study, density was measured at atmospheric conditions using pressurized mud balance from OFITE. The retort test was conducted to measure the oil/water contents, which is a method used to calculate the percentages of oil, water and solids. The test was conducted using 10 mL retort kit provided by FANN.

#### 2.2.2. Rheology Test

Drilling fluid serves a variety of purposes, including maintaining hydrostatic pressure, transporting and suspending drill cuttings. Drilling fluid rheology is one of these features. It is a branch of physics concerned with the flow and deformation of materials. It can assist us in comprehending and explaining fluid behavior under a variety of variables, such as temperature, pressure and external forces. Plastic viscosity, yield point and gel strength are all rheological properties of drilling fluids. The ability to track mud rheological parameters under downhole circumstances is a critical aspect in determining the success of any wellbore drilling operation.

In this study, rheology test was conducted at 49 °C (120 °F) temperature and atmospheric pressure conditions using a digital viscometer from OFITE (Model 900). Plastic viscosity (PV) and yield point (YP) were calculated using Equations (1) and (2):

(1) $$\mathrm{PV}\;\left(\mathrm{cP}\right)=\emptyset_{600\mathrm{rpm}}-\emptyset_{300\mathrm{rpm}}$$

(2) $$\mathrm{YP}\;\left(\frac{\mathrm{lb}}{100{\mathrm{ft}}^{2}}\right)=\emptyset_{300\mathrm{rpm}}-\mathrm{PV}\;\left(\mathrm{cP}\right)$$

The gel strength was determined as per the procedure reported in our previous publication [46].

#### 2.2.3. Fluid Loss Test

Filtration tests are a method for evaluating a drilling fluid’s filtration behavior and wall cake-building features. They inform how much drilling fluid filtrate has infiltrated a porous and permeable formation. Further, it indicates the change in the degree of wettability alteration in the near-wellbore zone due to filtrate invasion. It shows the potential for the tight hole or differential sticking problem.

The loss of liquid (filtrate) from a drilling fluid due to filtration is controlled by the filter cake formed of the solid constituents in the drilling fluid. The test in the laboratory consists of measuring the volume of liquid forced through the filter paper for a 30-min period under given pressure and temperature using an HPHT fluid loss cell. The drilling fluids’ fluid loss and wall building potential were determined by applying the API standard procedure (API-RP-13B) [17]. The fluid loss test was conducted at 3.45 MPa (500 psi) differential pressure and at 177 °C (350 °F) temperature for a period of 30 min using OFITE HPHT fluid loss tester [46]. The filtrate was collected for 30 min in a graduated cylinder. The thickness of the filter cake was measured using a digital Vernier caliper.

#### 2.2.4. Electrical Stability Test

An electrical stability test is performed to evaluate the drilling fluids’ emulsion stability and oil-wetting qualities [47]. The test was conducted at 49 °C (120 °F) temperature using electrical stability (ES) tester from FANN. The ES probe is put into a drilling fluid-filled cup and the test is completed by pressing a test button. The ES meter automatically applies an increasing voltage across an electrode gap in the probe (from 0 to 2000 V). The electrical stability of the drilling fluid is the maximum voltage that it can withstand across the gap before a current break-through [25].

#### 2.2.5. Zeta Potential Test

Zeta potential (ζ) is defined as the difference between the potential of the dispersion medium and the stationary layer of liquid adhering to the particles. It is a vital particles physical property that determines the coagulation and dispersion of the particles in the dispersion medium. Further, the colloidal stability and surface modifications are studied using zeta potential measurements [48]. The wettability of the surface can be quantitatively determined by zeta potential measurement. In this study, barite particles suspension was studied using zeta potential. The solution was prepared by mixing 40 mg of barite in 30 mL of distilled water. The solution was stirred for 24 h. At the end of 24 h, the ζ-potential was measured at 22 °C (72 °F) temperature. After establishing the base sample, two drops of oil wetter were added to the base solution and stirred for 24 h. At the end of 24 h, the ζ-potential was measured and compared with the base case without the oil wetter.

## 3. Results and Discussion

### 3.1. Density and Oil/Water Contents

**Figure 1:**
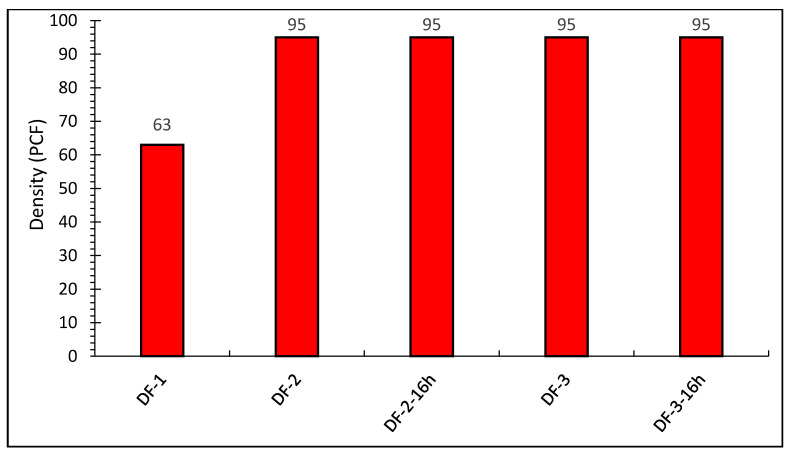
The density of OBDF drilling fluids.

The drilling fluid formulation DF-1 had a density of 63 PCF, whereas DF-2 and DF-3 had densities of 95 PCF mainly caused by the added barite. The formulations’ densities were steady at 95 PCF even after hot rolling as shown in Figure 1 indicating that the hot rolling (aging) went well, no leakage and no water vapours escaped from the mud, which could have influenced the mud density. Unit conversions from oilfield units to SI units are provided in Appendix A.

A retort test was conducted to determine the oil-water contents in the drilling fluids. The oil water content in DF-2 and DF-3 differed slightly from DF-1 because barite increased the percentage of solids and decreased the percentage of liquids. For DF-1, the oil content is 81% and water content is 19%. The oil-water content of DF-2 and DF-3 were nearly the same (82% oil content and 18% water content). After hot rolling, no noticeable changes in the oil water contents were observed. DF-4 had a slight change in oil-water contents from DF-3. In DF-4, water volume was slightly reduced and the solid percentage was slightly increased compared to DF-3 by adding more fluid loss control additive.

### 3.2. Rheology

Rheological properties such as yield point, plastic viscosity and gel strength were measured. The resistance of a fluid to flow is referred to as plastic viscosity. It is used to determine the size, shape, distribution and number of solids, as well as the liquid phase’s viscosity. It is undesirable to have a fluid with a high plastic viscosity as high viscosity increases the equivalent circulating density (ECD) and energy required to run the pumps. The plastic viscosity of DF-1 was 18 cP, while the addition of barite increased the plastic viscosity of DF-2 by 106.1% to 37.1 cP. For DF-3, the addition of rheology modifier and oil wetter increased the PV by 10.8% to 41.1 cP. Furthermore, the PV was affected by hot rolling at the high temperature similar to the behaviour observed by previous studies [46]. Although, after 16 h of hot rolling, only a relatively slight decrease in PV was observed (9.97% decrease for DF-2 and 3.16% decrease for DF-3), which indicates the stability of the drilling fluids. Further increase in the hot rolling period to 32 h reduced the PV of DF-2 by 23.18% to 28.5 cP and DF-3 by 9.73% to 37.1 cP when both are compared to the drilling fluids before hot rolling. Therefore, DF-3 formulation showed better stability (Less decrease in PV) after hot rolling when compared to DF-2. The concentrations of some additives in DF-3 were adjusted and formulated DF-4. DF-4 was hot rolled for 32 h. After hot rolling, the PV was slightly reduced to 35.8 cP, which is very close to the PV of DF-3.

**Figure 2:**
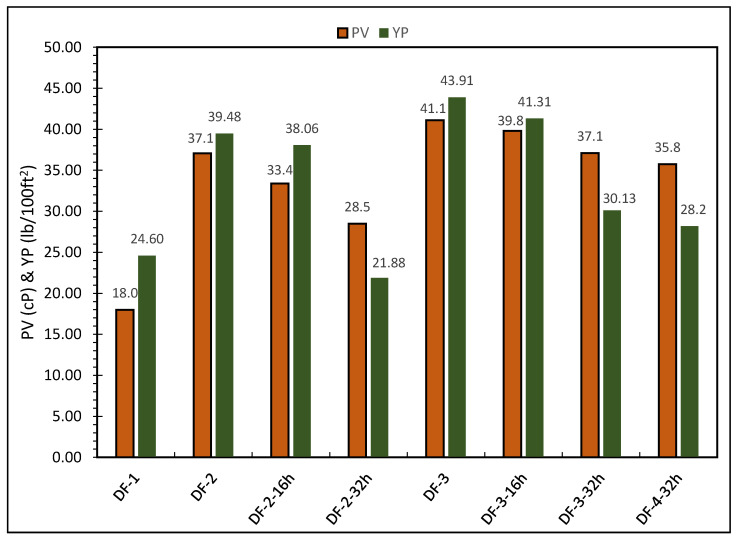
The plastic viscosities and yield points of the OBDFs.

The yield point is the stress required to initiate fluid movement and represents the resistance to beginning flow. If the applied stress is less than the yield stress, the fluid will regain its strain when the applied stress is removed. Electrical charges on or near the surface of the particles cause this resistance. Higher yield points result in higher frictional pressure losses and an increase in ECD. For the yield point, DF-1 showed a yield point of 24.6 lb/100 ft^2^ while the yield point of DF-2 formulation having barite was 39.5 lb/100 ft^2^, which is 60.56% higher than DF-1. Further incorporation of rheology modifier and oil wetter increased the yield point by 11.16% to 43.91 lb/100 ft^2^. After hot rolling DF-2 for 16 h, the yield point was slightly decreased and it was found to be 38.1 lb/100 ft^2^. Therefore, DF-2 relatively maintained yield point after 16 h of hot rolling. After hot rolling for 32 h, the yield point of the formulation DF-2 decreased by 44.55% —when compared to prior to hot rolling—to 21.9 lb/100 ft^2^. This shows that DF-2 lost rheology after 32 h of hot rolling. For DF-3, similar behaviour of DF-2 was observed but with lighter impact on yield point after 32 h of hot rolling. The yield point of DF-3 decreased by 31.38% from 43.91 to 30.13 lb/100 ft^2^ after 32 h of hot rolling. For DF-4, the yield point was decreased to 28.18 lb/100 ft^2^ after 32 h of hot rolling. It was observed that prolonged high temperature aging affected the rheology of drilling fluids. Figure 2 shows the PV and YP results for all the drilling fluids formulations.

**Figure 3:**
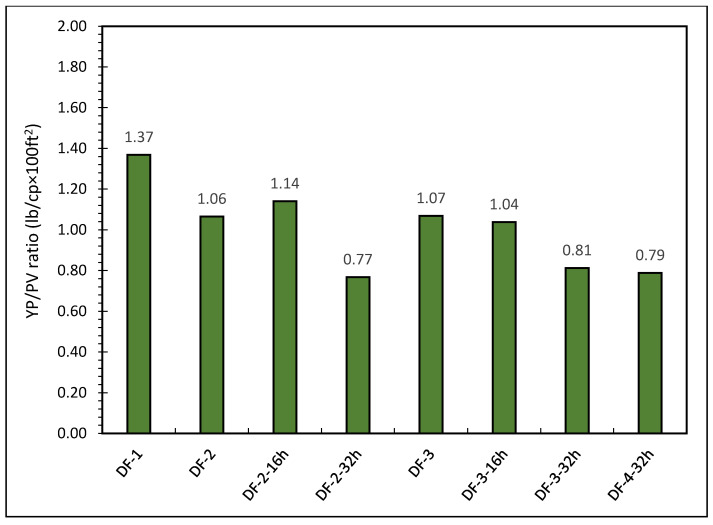
The YP/PV ratios of oil based drilling fluids.

The ratios of yield point to plastic viscosity (YP/PV) is used to investigate the cutting suspension of drilling fluids. The drilling fluids having a ratio in the range of (0.75–1.5) shows good suspension capacity. This ratio was highest for DF-1 was 1.37 while the YP/PV ratio for DF-2 formulation was 1.06. For DF-2, after hot rolling for 16 h and 32 h, the YP/PV ratio was 1.14 and 0.77, respectively. Therefore, after 16 h of hot rolling, the YP/PV ratio slightly increased by 7.55% and resulted in better suspension capacity of the drilling fluid. On the other hand, drilling fluid formulation DF-2 lost cuttings suspension capacity after 32 h of hot rolling as the YP/PV ratio decreased to 0.77. For DF-3, the YP/PV ratio was affected by hot rolling and it was decreased with high temperature exposure. A very slight change observed after 16 h hot rolling while after 32 h, the YP/PV ratio reduced to 0.81. Therefore, DF-3 showed better carrying capacity after 32 h of hot rolling when compared to DF-2. Furthermore, DF-4 after 32 h of exposure did not bring major change in its ratio when compared to DF-3-32h. The ratios of yield point to plastic viscosity (YP/PV) for the tested drilling fluids are shown in Figure 3.

Gel strength describes the drilling fluid’s ability to suspend drill cuttings at static condition. The shear stress of a drilling fluid is measured with a viscometer at a low share rate (3 RPM) after being static for a length of time [10 s and 10 min in the API standard]. For the gel strength, 10 s and 10 min gel strengths were measured for all formulations. The 10-s and 10-min gel strengths for DF-1 formulation were 10 and 11 lb/100 ft^2^, respectively. For DF-2, the gel strength for 10 s and 10 min were the same at 20 lb/100 ft^2^. After hot rolling DF-2 formulation for 16 h, the gel strength for 10 s and 10 min were 20 lb/100 ft^2^ and 23 lb/100 ft^2^, respectively. It showed that DF-2 maintained gel strengths after 16 h of hot rolling but after 32 h of hot rolling, DF-2 gel strengths for 10 s and 10 min were decreased significantly to 10 lb/100 ft^2^ and 12 lb/100 ft^2,^, respectively. The addition of rheology modifier and oil wetter did not bring much change in gel strength before hot rolling as noticed in DF-3. After hot rolling for 16 h, 10-s and 10 min gel strengths for DF-3 increased to 20 and 30 lb/100 ft^2^ from 17 and 19 lb/100 ft^2^ respectively. After 32 h of hot rolling, slight reduction in gel strengths was noticed compared with gel strength after 16 h, but the values are still high compared to gel strength before hot rolling.

**Figure 4:**
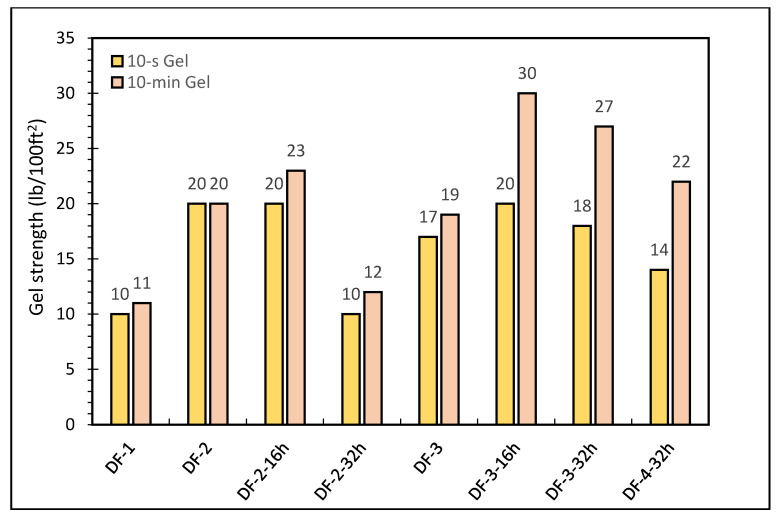
The gel strengths of oil-based drilling fluids.

Similarly, a reduction in gel strength is observed in DF-4 after 32 h hot rolling. For DF-4, the values of 10-s and 10 min gel strengths were less when compared to DF-3 after 32 h of hot rolling. Marinating the gel strength even after 32 h of hot rolling was mainly due to the rheology modifier and the oil wetter. The gel strength results of the different drilling fluid formulations are given in Figure 4.

From the rheology results, it can be inferred that DF-2 and DF-3 maintained their rheology profile after 16 h of hot rolling (less than 10% decrease for DF-2 and DF-3 in both PV and YP). Therefore, the flat rheology profile was obtained after 16 h of hot rolling. However, when hot rolled for 32 h, the rheological properties of DF-2 drastically changed (23.18% decrease in PV and 44.55% decrease in YP). Therefore, DF-2 lost its flat rheology profile. On other hand, DF-3 was affected less (less than 10% decrease in PV and 31.38% decrease in YP) when compared DF-2. Therefore, DF-3 only slightly lost its rheological profile after 32 h hot rolling when compared DF-2.

Furthermore, DF-4 reduced rheological parameters after 32 h hot rolling and maintained results close to DF-3-32h. This showcases the impact of the rheology modifier and oil wetter additives in obtaining flat rheology of drilling fluids. The PV was maintained in DF-3 and DF-4 due mainly to the addition of the oil wetter. It is apparent that the oil wetter made the calcite and barite particles more oil-wet and therefore enhanced the stability and suspension of calcite and barite in oil [49].

### 3.3. HPHT Fluid Loss

For the HPHT fluid loss tests, DF-1 formulation had less filtration loss than the DF-2 and DF-3 formulations, but with minor difference. The fluid loss was directly affected by the weighed material. However, after hot rolling DF-2 and DF-3 for 16 and 32 h, the fluid loss dramatically increased. After hot rolling for 16 and 32 h, fluid loss of DF-2 increased by 106% and 446%, respectively. Similarly, for DF-3, fluid loss increased after hot rolling for 16 and 32 h by 144% and 450%, respectively. After 16 h of high temperature aging, the emulsion stability improved, but the fluid loss volume increased. It showed that the emulsifiers retained their properties after 16 h high-temperature aging, while the fluid loss controller lost it. The most likely reason of increased fluid loss volume is the performance and quantity of fluid loss control additive. DF-4 was mixed with some changes to specific additives to keep the qualities of DF-3 at high temperatures. The amount of fluid loss control additive in DF-4 was raised among the adjusted additives. After 32 h of hot rolling, the increase in fluid loss control additive quantity had a favorable effect on fluid loss and sustained fluid loss at 2.5 mL. From the fluid loss experiments on the different drilling fluids, it was observed that the formulation of additives used in DF-4 has prevented the sag of the solids (barite and calcite) after extended period of hot rolling and in turn reduced the filtration (fluid loss is 2.5 mL only). This formulation with the additives used, such as the oil wetter, greatly reduces the sag issues in oil-based mud.

**Figure 5:**
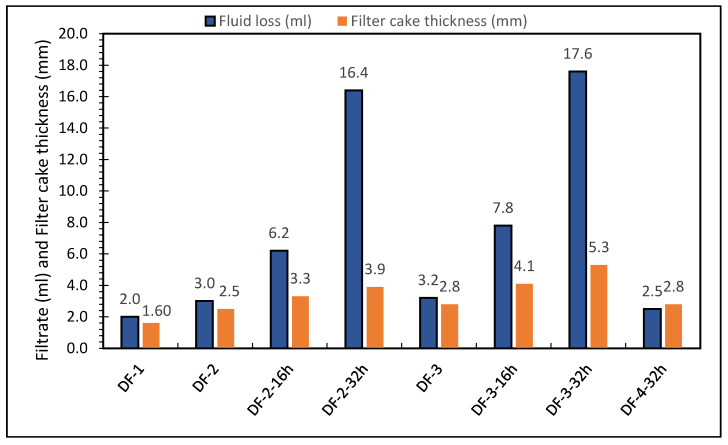
Fluid losses and filter cake thickness of filters obtained from of oil-based drilling fluids.

The filter cake thickness was measured for all the drilling fluid formulations and it was observed to increase in direct relation to fluid loss. A thin filter cake was obtained from formulation DF-1 (1.6 mm) and a thick filter cake was obtained from DF-2 and DF-3 after 32 h of hot rolling (3.9 mm and 5.3 mm, respectively). After an extended period of hot rolling, DF-4 still resulted in thin filter cake with respect to its fluid loss at high temperature conditions. Having a thin impermeable filter cake will help maintain the hole and allow subsequent operations to run smoothly. Using the OBDF formulation with the additives used in DF-4 achieved this thin impermeable filter cake even after going through an extended period of hot rolling. Figure 5 shows the results of the fluid loss tests on all the drilling fluid formulations and provides filter cake thickness values for tested drilling fluids. Overall, the filter cake thickness increased with respect to fluid loss volume.

### 3.4. Electrical Stability

To investigate the emulsion stability of the drilling fluids, electrical stability (ES) test was carried out at a temperature of 49 °C (120 °F). The higher electrical stability voltage the better is the emulsion stability. Before hot rolling, formulation DF-3 had the highest electrical stability (609 V) compared to formulation DF-1 (288 V) and DF-2 (560 V). It demonstrated the combined effect of barite, rheology modifier and oil wetter in improving the drilling fluid’s emulsion stability. Later, DF-2 and DF-3 were hot-rolled for 16 h and 32 h. After each hot rolling, emulsion stability was measured. It was observed that after hot rolling for 16 h, the voltage value increased by 19.28% to 668 V from 560 V for DF-2. For DF-3, it increased by 10.50% to 673 V from 609 V. This increase in voltage shows a higher emulsion stability of the DF-2 and DF-3 after 16 h of hot rolling. However, increasing the hot rolling period to 32 h resulted in lower voltages and reduced emulsion stability. This is similar the behaviors observed in previous relative studies [46,48]. After 32 h of hot rolling, DF-2 drilling fluid lost emulsion stability as the voltage value decreased by 42.67% to 321 V. A similar scenario is seen in DF-3 and DF-4 although with less impact as the voltage value of DF-3 decreased by 26.76% to 446 V after hot rolling for 32 h and DF-4 reached 360 V. The extended aging at high temperatures, as seen in all formulations, has a significant impact on emulsion stability. The DF-2 drilling fluid was the most affected, as it lost its emulsion stability. In DF-3 and DF-4, a similar response was reported, albeit with less impact. It was primarily maintained due to the oil wetter and rheology modifier.

**Figure 6:**
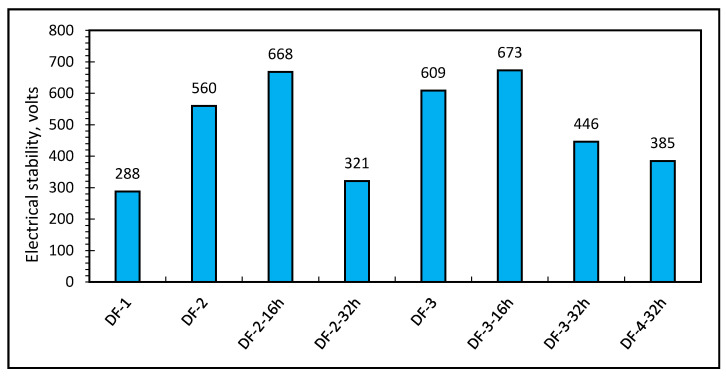
Electrical stabilities of oil-based drilling fluids.

Given that the recommended electrical stability is to be more than 400 V, DF-3 maintained an electrical stability above 400 V even after 32 h of hot rolling. DF-4 showed lower than 400 V (385 V) electrical stability—after 32 h of hot rolling, which is not that far off from the recommended value especially when compared with the effect of extended period of hot rolling in DF-2. Figure 6 shows the electrical stability results for all the drilling fluid formulations.

### 3.5. Zeta Potential

The zeta potential test was conducted on two different samples. In the first sample, barite was mixed in water. In the second sample, the oil wetter was added in the first sample and investigated the change in wettability by measuring the zeta potential. It was observed that barite particles in distilled water resulted in negative zeta potential (−12.156 mV) at a pH of 7.4. The negative value of zeta potential depicts a water wet surface [50,51]. The addition of oil wetter reversed the zeta potential and made it positive (28.72 mV) with low standard deviation. The reversal in zeta potential from negative to positive could be due to destruction of double diffused layer.

Table 3 provides the zeta potential results with low standard deviation. Further, a high positive charge indicates an oil wetting surface [48]. The oil wetter basically changed the wettability from hydrophilic to hydrophobic by interacting and adsorbing on barite particles. This change in wettability of barite particles plays significant role in their suspension in oil-based drilling fluids and would prevent the barite sag problem. The zeta potential results explain the impact of the oil wetter in maintaining PV values as the oil wetter made the barite particles more oil wet enhancing the stability and suspension of barite in oil. A few researchers discussed modifying the surface of weighting material and altering it from water wet to oil wet. For instance, Massam et al. [49] discussed modifying the surface of barite from water wet to oil wet for its dispersion and suspension in the oil phase. This area is in development and yet to be explored under HPHT conditions for an extended period.

### 3.6. Environmental Impact

OBDFs are, in general, considered toxic to the environment, specifically to the marine environment. Nonetheless, certain circumstances require the use of OBDFs due to their inherent advantages—for example, drilling in shale formations, high-temperature stability, extended-reach or horizontal wells, high penetration rates, lubricity, low mud weight, prevention of hydrate formation, reduced waste generation and wellbore stability. Additionally, the high cost of OBDFs can be offset by enhanced drilling performance. OBDFs have the potential to reduce the environmental impacts through rapid drilling operations and low air emissions from rig machinery. The primary source of concern in OBDFs is the base oil, which is frequently diesel. Diesel oil is discouraged and is being phased out in favor of mineral oil or other synthetic oil to mitigate the environmental impact of OBDFs. The study’s primary objective is to demonstrate the value of oil wetter in drilling fluids, which is a surfactant with minimal environmental impact. Other chemicals used in the drilling fluids’ formulations are widely used as drilling additives worldwide.

## 4. Conclusions

Several experiments were conducted to assess the performance of four OBDF formulations (DF-1, DF-2, DF-3 and DF-4), including density, rheology, fluid loss, zeta potential and electrical stability studies. DF-1 is the original barite-free drilling fluid formulation. For DF-2, barite has been added as a weighting material. For DF-3, along with the addition of barite, rheology modifier and oil wetter were mixed too. DF-4 was mixed based on the performance of the DF-3 and was slightly modified from DF-3. DF-4 contained high concentrations of fluid loss controller and oil wetter. The water content was also reduced in DF-4. The rheology of the drilling fluids was changed by hot rolling at HPHT conditions. After 16 h of hot rolling, the rheology profiles of DF-2 and DF-3 were flat. While after 32 h of hot rolling, these formulations lost their rheological character, having a greater impact on DF-2 than DF-3 and DF-4. Clearly, the highest fluid loss gained was in drilling fluid that has been hot rolled for 32 h. Although, DF-4 has been hot rolled for 32 h, the increase in fluid loss control additive quantity had a favorable effect on fluid loss and sustained fluid loss at 2.5 mL. On the basis of experimental investigations, the following conclusions have been reached:

(1) The rheology of the drilling fluids was changed by hot rolling at HPHT conditions. After 16 h of hot rolling, the rheology profiles of DF-2 and DF-3 were flat. After 32 h of hot rolling, the formulations lost their rheological character, having a greater impact on DF-2 than DF-3 and DF-4. The oil wetter and rheology modifier positively impacted drilling fluid performance and provided flat rheology of drilling fluids.
(2) The fluid loss increased with the hot rolling duration, indicating that the fluid loss additive’s performance was compromised at high temperatures. Nevertheless, an increase in the concentration of the fluid loss controller assisted in maintaining the fluid loss as observed with DF-4 performance after 32 h of hot rolling.
(3) The formulation of additives used in DF-4 prevented the sag of the solids (barite and calcite) after an extended period of hot rolling and reduced the filtration (fluid loss is 2.5 mL only).
(4) The filter cake thickness was increased with respect to fluid loss volume. After an extended period of hot rolling, DF-4 still resulted in thin filter cake with respect to its fluid loss at high temperature conditions. Having a thin impermeable filter cake will help maintain the hole and allow subsequent operations to run smoothly.
(5) The oil wetter changed the barite particles’ wettability from hydrophilic to hydrophobic by interacting and adsorbing on barite particles which played a significant role in their suspension in OBDFs preventing the common barite sag problem.

## Figures and Tables

**Table 1:** Recommended range of rheological and fluid loss properties for OBDF [38,39,40].

Property	Range
Electrical stability, V	≥400 V
PV, cP	10–60 cP, preferably 15–40 cP
YP, lb/100 ft^2^	2.5–20, preferably 5–12.5
YP/PV ratio	0.75–1.50
Gels 10 s, lb/100 ft^2^	4–10
Gels 10 min, lb/100 ft^2^	4–15
HPHT Fluid loss °F, mL/30 min	<10
Filter cake thickness, 1/32 inch	2/32

**Table 2:** Formulations of the OBDFs.

Additives in Order of Mixing	Functions	Mixing Time (Minutes)	DF-1	DF-2	DF-3	DF-4
Diesel, mL	Continuous fluid	0	200	200	200	200
Primary emulsifier, g	Generates emulsion	5	8	8	8	8
Secondary emulsifier, g	Emulsion stability and wettbility	2	4	4	4	4
Lime, g	pH enhancer	10	6	6	6	8
Water, mL	Dispersed fluid	5	50	50	50	45
CaCl_2_, g	Shale stabilizer		32	32	32	32
Viscosifier, g	Impart viscsoty	10	10	10	10	10
Fluid loss controller, g	Fluid loss reduction	5	7	7	7	11
CaCO_3_, g	Bridging agent	10	30	30	30	30
Barite, g	Weighting material	10	-	200	200	200
Rheology modifier, g	Improve rheology	5	-	-	2	2
Oil wetter, g	Wettability alteration	5	-	-	0.5	1

**Table 3:** Zeta potential results.

Solutions	Zeta Potential	Standard Deviation
	mV	mV
Barite + water	−12.156	1.052
Barite + water + oil wetter	28.72	0.604

## Data Availability

The data presented in this study are available within the article.

## Sample Availability

Samples of the compounds are not available from the authors.

## Abbreviations

PV	Plastic Viscosity/ cP
∅	Viscometer dial reading at different speeds, dimensionless
YP	Yield Point: lb/100 ft^2^
ζ	Zeta Potential: mV

## Appendix A

**Table A1:** Unit Conversions.

Property	Oilfield Units	SI Units	Conversion Factor
Shear rate	RPM	1/s	1/s = 1.703 × RPM
Plastic viscosity	cP	mPa⋅s	1 mPa⋅s = 1 cP
Yield point	lb/100 ft^2^ or dynes/cm^2^	Pa	1 Pa = 10 dynes/cm^2^
Density	PCF	kg m^−3^	1 kg m^−3^ = 0.062 PCF
Fluid loss	mL	m^3^	m^3^ = 100,000 mL
Filter cake thickness	1/32 inch	mm	1 mm = 0.0393 inch
